# HIV and the eye

**Published:** 2020-03-30

**Authors:** Stephen Gichuhi, Simon Arunga

**Affiliations:** 1 Consultant Ophthalmologist & Senior Lecturer: Department of Ophthalmology, University of Nairobi, Kenya.; 2 Consultant Ophthalmologist & Lecturer: Department of Ophthalmology, Mbarara University of Science and Technology, Uganda.

**People with poorly managed HIV infection have an increased risk of eye problems, including microbial keratitis, adverse drug reactions and tumours of the eye.**

The main ocular effects of HIV are related to immune suppression and impaired immune surveillance of tumours. HIV compromises cell-mediated immunity, thereby increasing the risk of infection with:

- bacteria (e.g., those causing tuberculosis and syphilis)
- fungi (e.g., *Candida spp.* and *Cryptococcus spp.*)
- parasites (e.g., *Toxoplasma gondii*)
- viruses (e.g., herpes zoster virus, human papillomavirus, Kaposi sarcoma-associated herpes virus, cytomegalovirus and Epstein-Barr virus).

Patients with lower CD4 counts are more likely to have ocular manifestations^1^; however, use of antiretroviral therapy (ART) has modified the epidemiology of ocular manifestations and variations in the predominant subtype of HIV may also lead to geographical differences in eye disease.

## Anterior lesions

### Herpes zoster ophthalmicus (HZO)

**Figure 1:**
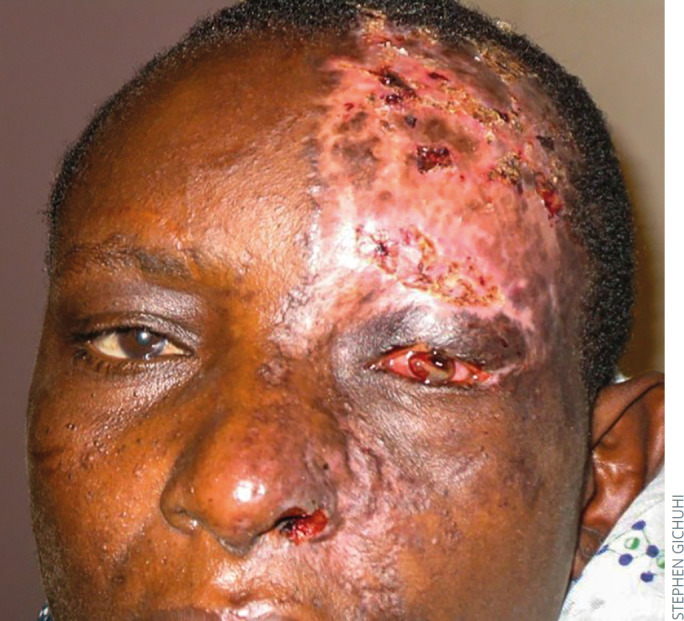
Herpes zoster ophthalmicus with corneal ulcer perforation

HZO is caused by reactivation of latent varicella zoster virus in the trigeminal ganglion; this is covered in detail on pp. 71–72. Within the context of HIV infection, it tends to present more severely, with a painful vesiculo-bullous rash that follows the distribution of the ophthalmic branch of the trigeminal nerve on one side of the face, without crossing the midline. In the acute phase, there is usually swelling of the eyelids. Extension of the rash on the side of the nose, due to involvement of the nasociliary nerve (Hutchinson's sign), is often associated with intraocular inflammation and corneal denervation. This can lead to corneal ulceration and iritis (Figure 1). The rash heals with scarring and may be complicated by post-herpetic neuralgia. Reduced corneal sensation as a sequelae of HZO increases the risk of neurotrophic keratopathy, persistent epithelial defects, microbial keratitis and, ultimately, corneal scarring.

**“The main ocular effects of HIV are related to immune suppression and impaired immune surveillance of tumours.”**

The diagnosis is usually based on clinical signs. See Table 1 (p. 78) for treatment guidelines.

### Molluscum contagiosum

**Figure 2:**
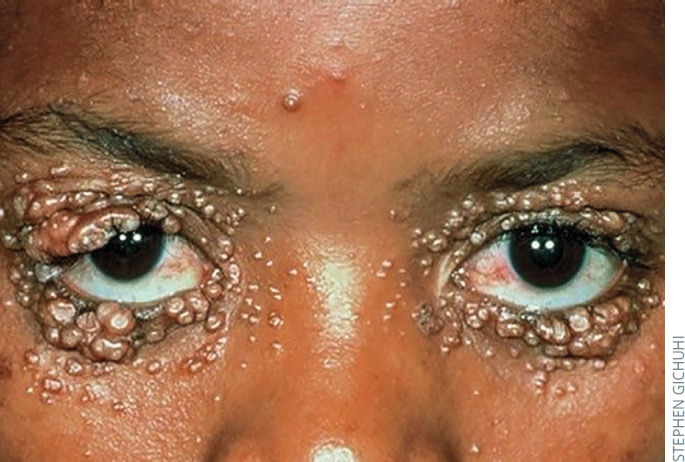
Molluscum contagiosum

Molluscum contagiosum virus belongs to the pox family of viruses. It causes raised umbilicated skin nodules which, in HIV patients, can be widespread. (Figure 2). The diagnosis is clinical, and management involves curettage with or without cryotherapy.

### Keratoconjunctivitis sicca (dry eyes)

Dryness of the ocular surface (not due to Sjögren's syndrome) has a reported prevalence of 11–50% among individuals with HIV/AIDS. Long-term use of ART and vitamin A deficiency are believed to be the main contributors.^2^ Ocular lubricants are helpful in relieving symptoms.

**Figure 3:**
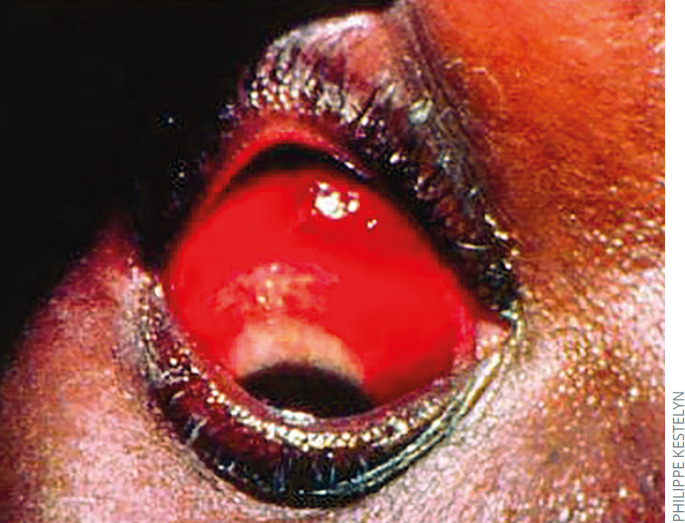
Kaposi's sarcoma

### Kaposi's sarcoma

Kaposi sarcoma appears as reddish-purple vascular tumours on the conjunctiva or lid margin. (Figure 3). The incidence has reduced with ART use. Various treatments have been advocated, including local excision, focal radiotherapy, intralesional vinblastine, alpha interferon or liposomal daunorubicin.

**Figure 4:**
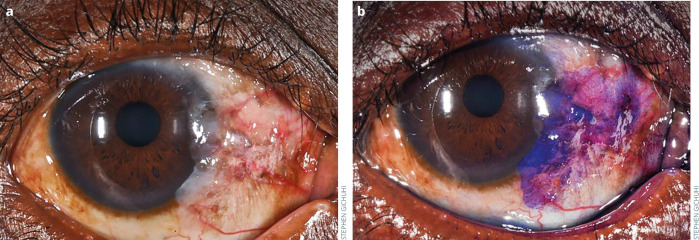
Ocular surface squamous neoplasia (OSSN) before (a) and after toluidine blue vital staining (b)

### Ocular surface squamous neoplasia (OSSN)

OSSN is a spectrum of tumours ranging from intraepithelial neoplasia to invasive squamous cell carcinoma. In Africa and Asia, most patients are young (<40 years). Africa saw a dramatic rise in incidence after the HIV pandemic started. HIV is the main risk factor, particularly in those who do not take ART. Other risk factors include exposure to ultraviolet radiation, human papilloma virus, albinism, xeroderma pigmentosum, allergic conjunctivitis and possibly cigarette smoking. Histopathology provides a definitive diagnosis. Toluidine blue 1% vital staining is a useful tool for marking the extent of tumour, guiding the excision boundaries; and for early detection of recurrences after treatment.^3^ (Figures 4a and 4b)

Excision is the mainstay of treatment. Adjuvant therapies include cryotherapy, antimetabolites (5FU^4^ and Mitomycin C), and radiation. For orbital spread, external beam radiotherapy and/or exenteration may be considered. Primary therapy with antimetabolites and interferons is gaining popularity but is lacking in robust clinical evidence at present.

### Microbial keratitis

Microbial keratitis, can be caused by bacteria, viruses, protozoa, and fungi. It is characterised by eye pain, conjunctival hyperemia and corneal ulceration with a stromal inflammatory cell infiltrate. HIV-positive patients are at higher risk of developing microbial keratitis, which is usually characterised by rapid progression, slow response to treatment and poor outcomes.^5^ Treatment focuses on identifying the causative agent and starting effective antimicrobial therapy. A detailed management approach has been described in previous issues of this journal.^6^ For fungal keratitis, natamycin 5% is currently the preferred option for filamentous fungi, whilst amphotericin B is the drug of choice for candida infections. Topical antibiotics remain the best treatment for bacterial keratitis, with good response to fluoroquinolones, aminoglycosides and cephalosporins, depending on the local antimicrobial susceptibility patterns.

### Uveitis, including immune reconstitution uveitis

Uveitis in HIV patients often has an infectious aetiology, with the most common causes being herpes simplex virus and the bacteria responsible for tuberculosis and syphilis. Treatment includes topical, sub-Tenon's or intravitreal steroids, cycloplegia, and antimicrobial therapy for the underlying infection.

Immune reconstitution uveitis (IRU) presents with vitritis, cystoid macula oedema, epiretinal membranes, angiitis, papillitis or neovascularisation. It was first described in AIDS patients with CMV retinitis whose CD4 lymphocyte count rose from ≥50 cells/µL to ≥100 cells/µL after starting highly active antiretroviral therapy (HAART). It is not common in non-CMV eyes and may be due to CMV infection breaking the blood-ocular barrier, thereby exposing the CMV antigen to an improved T-lymphocyte response. Treatment of IRU involves intravitreal steroids and anti-CMV therapy.

**“Cataract occurs earlier in HIV-infected patients, possibly because HIV causes early biological ageing.”**

### Cataract

Cataract occurs earlier in HIV-infected patients, possibly because HIV causes early biological ageing.^7^ Cataract surgery in these patients is safe and effective.

## Posterior segment lesions

### HIV retinopathy

This microvasculopathy presents with transient cotton wool spots and does not cause visual loss. No treatment is needed.

### Cytomegalovirus (CMV) retinitis

*See article on pp. 79–80 of this edition*

### Progressive outer retinal necrosis

This is a very aggressive necrotising disease of the retina caused by varicella zoster virus (VZV), HSV 1 or 2, or CMV. It is associated with extreme immunosuppression (CD4 <50 cells/ml). See Table 1 for treatment.

### Toxoplasmosis

Retinochoroiditis caused by *Toxoplasma gondii* has an atypical presentation in HIV positive patients, displaying more fulminant disease with multifocal lesions, more vitritis (‘headlight in the fog’ appearance), bilateral disease, orbital cellulitis, neuro-retinitis and association with central nervous system disease, especially with lesions near the optic disc. Treatment for ocular toxoplasmosis should be given in consultation with a physician as part of the management of the systemic HIV infection. The possible treatment regimens are:

- Pyrimethamine (avoid in pregnancy or if breastfeeding) with folinic acid (to minimise bone marrow toxicity of pyrimethamine) in combination with sulfadiazine (or clindamycin)
- Azithromycin is a possible alternative to the above treatment
- Prednisolone may be considered once the infection is under control.

**Figure 5:**
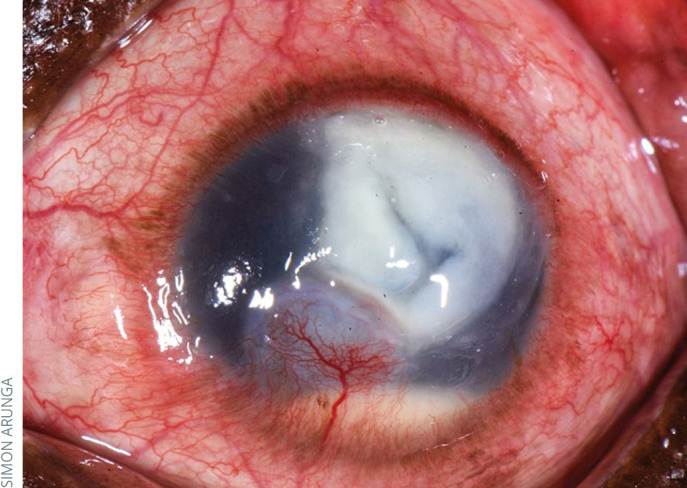
Candida microbial keratitis with hypopyon

## Neuro-ophthalmic disease

Various neuro-ophthalmological disorders occur in people living with HIV, including ocular motility disorders and palsies, visual field defects and optic neuropathy. Papilloedema also occurs secondary to cryptococcal meningitis, tuberculous meningitis, central nervous system toxoplasmosis and neurosyphilis. A unique neuro-retinal disorder was described in a US study as manifesting with decreased contrast sensitivity, abnormal perimetry and loss of retinal nerve fibre layer, associated with increased mortality.^8^

## Adverse drug reactions

Stevens-Johnson syndrome and toxic epidermal necrolysis (SJS/TEN) is an acute inflammatory vesiculobullous reaction of the skin and mucous membranes that also presents with marked bilateral conjunctival injection, discharge, chemosis and symblepharon. The patient looks like she or he has burns. The exact pathophysiology is not understood. In Africa, a high percentage of SJS/TEN patients are HIV positive and the majority will develop chronic ocular complications such as symblepharon, trichiasis, subconjunctival fibrosis, corneal scarring and vascularisation. SJS/TEN may be precipitated by drugs (e.g., sulfonamides and other antibiotics, anticonvulsants, isoniazid and – of particular relevance in the context of HIV – antiretroviral drugs).

Treat using topical steroids, with supportive measures such as ocular lubrication and pain control. If indicated and available, intravenous immunoglobulin G with plasma exchange may be considered. Scleral lenses can be used to prevent symblepharon, and mucous membrane grafts may be indicated.

## Conclusion

Although provision of ART and cotrimoxazole prophylaxis are subsidised worldwide, HIV-related ocular diseases remain an important cause of visual impairment.

**Table 1:** Guidelines for treatment of HIV-related ocular diseases discussed in this article

**Condition**	**Treatment** Ophthalmic treatment should form part of a multi-disciplinary strategy with infectious disease or HIV specialists, particularly for systemic treatment, including ART
**HZO**	Antivirals e.g., aciclovir or famciclovir (either intravenously or orally: check renal function and give appropriate dose for weight) For iritis, use topical steroids and cycloplegics Treat high IOP (e.g., with Gutt Timolol 0.5% twice a day). (If IOP > 30 mmHg, use oral acetazolamide 250mgs four times a day for 7-10 days) *See article on pp. 71–72.*
**CMV retinitis**	*See article on pp. 79–80*
**Progressive outer retinal necrosis**	Aciclovir intravenous (IV) initially, then orally for 6 weeks
**Toxoplasmosis**	Pyrimethamine (avoid in pregnancy or if breastfeeding) Folinic acid to minimise bone marrow toxicity of pyrimethamine Clindamycin with sulfadiazine Azithromycin monotherapy Prednisolone orally may be considered
**SJS/TEN**	Topical steroids, with supportive measures such as ocular lubrication, scleral lenses to prevent symblepharon, mucous membrane grafts and pain control Intravenous immunoglobulin G with plasma exchange.
